# Correspondence

**Published:** 1869-03

**Authors:** Thomas W. Evans

**Affiliations:** Surgeon Dentist to the Emperor.


### CORRESPONDENCE.

### ARTICLE V.

15 rue de la Paix.
Paris, *Jan.* 20, 1869.

*To the Editors of the American Journal of Dental Science.*

Sirs :—

I am induced to make the present comunication to the *American Journal of Dental Science*, from having seen in several publications—English and American—statements more or less erroneous concerning the introduction of Nitrous Oxide into dental practice in France and England.

Perhaps no questions are more difficult of solution than those involving rights of discovery or claims to a priority of use. If the points at issue were simply personal, the public could scarcely be interested in their discussion. The moment however a new and valuable discovery or application is made, it becomes a part of the world's property, and as such is entitled to its own history. Facts viewed simply as facts are seldom uninteresting, and there is generally among men so considerable a regard for historic accuracy, as well as so large a love of justice, that the correction of errors is often even less a matter of private satisfaction than of public interest.

In 1863, the attention of the Dental profession in the United States was directed—after an interval of nearly twenty years—to a reconsideration of the anæsthetic value of Nitrous Oxide. Experimental trials were followed by favorable reports, the use of the gas rapidly became popular, and nitrous oxide was soon generally regarded as not only the most agreeable, but as the safest, and best known agent, for the production of anæsthesia in dental operations. American testimony on that point seemed conclusive, and yet until quite recently there has been no reputable dentist in Europe, who has possessed either sufficient initiation or sufficient confidence in the merits of the gas, to even make with it an experimental trial, I make this statement advisedly. Some four years since the gas was administered by a gentleman in London, in a small number of cases, and a Frenchman in Paris, calling himself an "American Dentist," pretends to have employed the gas now three or four years. In the first instance, the trial was so imperfectly made that the gas was considered to be a failure. (see *British Medical Journal* of Nov. 7, 1868.) In the second case, the use of the gas by the individual referred to, could only have excited suspicion and distrust.

Convinced as I had been for a considerable time of the value of the gas, as well as of the advantages which might at once follow a more extended recognition of its merits, I determined it should have at least a full and fair trial on this side of the Atlantic. A circumstance afforded me an opportunity for carrying out my wishes. In the winter of 1865-6, I had sent an agent to the United States for the purpose of organizing a collection of hospital and sanitary material, which I proposed to exhibit at the Paris Exposition. Mr. Derby the United States Exposition agent at New York, one day in conversation with that gentleman, observed, that that Dr. J. Q. Colton had made application to him for space in the U.S. section for the exhibition of a Nitrous Oxide apparatus, and that the application had been refused by Mr. Beckwith the Commissioner at Paris. "Perhaps" continued Mr. Derby " you can take it."

The answer made was that as such an apparatus could hardly be classed with sanitary material, it would be necessary to refer the question to me, my reply was—" by all means bring it out." To this end an arrangement was shortly afterwards made with Dr. Colton. The apparatus, one of Sprague's, was sent to Paris, and was finally placed in the pavilion in the Champs de Mars, which contained my Sanitary collection, and there remained on exhibition during nearly the whole period of the Exposition. Gas was made with the apparatus every few days, and the physiological action of the gas, as well as the mode of preparing, were particularly shown to hundreds of surgeons who were attracted to the pavilion by the sanitary collection.

I also gave a number of public *seances* at my office, and in the presence of large numbers of scientific gentlemen, explained the special advantages of the new anæsthetic, which at the same time I exhibited experimentally. I subsequently presented the subject still more publicly, by calling it up at the meetings of several of the Medical societies of Paris, and by experimenting with the gas before committees appointed by these societies to investigate the subject.

I am sorry to say that these exhibitions although entirely successful and satisfactory to myself, failed to carry the conviction which I wished.

All sorts of so called practical objections were raised against substituting the gas for chloroform, and it was even greatly doubted if it was any safer or possessed any peculiar advantages over that, in Europe, almost universally employed anæsthetic agent. I was forced to content myself with having consciously directed to the subject the attention of some of the most eminent continental surgeons.

I continued however to use the gas in my own practice, and learning daily to appreciate it more highly, and to have more confidence in it, I determined to make another and more vigorous effort in its behalf.

Accordingly in April last, I went to London, for the express purpose of bringing the subject formally before the Odontological Society of that city. I gained a hearing, and more, an opportunity of administering the gas to a number of patients in the Dental and Ophthalmic hospitals—these administrations were most successful. The greatest interest was at once manifested by all the professional gentlemen who witnessed them. It seemed to be the beginning of a realization of what Horace Wells so pertinently and prophetically termed, " a new era in tooth pulling." It was but the beginning however, for scarcely had the success of my exhibitions ceased to be the subject of general personal congratulations, before a loud cry was heard from the camp of routinists.

At a meeting of the Medical Society of London, Dr. B. W. Richardson pronounced Nitrous Oxide to be " The best known, the least wonderful and most dangerous of all substances which had been applied for the production of general anæsthesia," and the "*Lancet*" added that " He had not spoken a moment too soon. The *ad captandum* method of applying the most potent medicinal agents against the teachings of scientific experiment and the experience of accepted observers is a phase in physic which requires to be put down with a strong hand." The "*Times and Gazette*," echoing the same sentiments, denied even the anæsthetic properties of nitrous oxide which it contemptuously called an "asphyxiating agent."

The friends of progress were however prepared, and were resolved that nitrous oxide before receiving a summary condemnation should at least receive a fair trial before an English jury. The *British Medical Journal* advocated the cause of the gas, guardedly. Communications giving the favorable results of trials, flowed in upon the London Medical Journals. Two or three startling deaths from chloroform in a single week in London, directed the attention of even the secular press to the new anæsthetic. And perhaps it may not be improper for me also to say, that desiring as I did to secure an extended and thorough trial, I gave to the Dental Hospital of London the sum of £100 to be expended in experimenting with the gas.

The result of the inquiry then instituted I have just learned, from the "First report of the joint committee appointed by the Odontological Society" to investigate the subject. The report has been carefully prepared, presents the disadvantages as well as the advantages peculiar to the anæsthetic use of the gas, and deals with conclusions cautiously. Two or three important facts are however evident to the committee.

The gas is a powerful anæsthetic.

It is as safe, probably safer, than any other anæsthetic.

It possesses over all other anæsthetics certain marked advantages.

These facts have induced the gentlemen who compose the committee to observe that they propose to *continue* to use the gas, and to suggest that it may finally become in England as popular an anæsthetic as it now is in the United States.

This expression of opinions is but just, supported as it is by the well known fact, of the very rapid introduction of the use of nitrous oxide gas into private dental and surgical practice not only in London, but in nearly all the cities of Great Britain. Probably no new substance or new application has ever been introduced into English dental practice more rapidly and at the same time more generally than this.

The successful introduction of the anæsthetic use of nitrous oxide into England has also produced its reaction upon the Continent. French, Belgian and German dentists are now begining to make use of the gas.

Mathien, Billard & Son and others, are at the present time supplying frequent orders for apparatus, and I have no hesitation in expressing my belief that within a comparatively short time the use of the gas will become as general in Europe as it now is in America.

The results so far have been eminently satisfactory. A great concession has been made, a recognition substantially expressed of that *leadership* which American dentistry has taken since the beginning of the century. But what is of still greater consequence, the use of dangerous anæsthetics will be limited and human life less frequently imperiled by the introduction into a new and wide field of practice of a simple, safe, and effective means of producing temporary insensibility to pain.

Such is a hasty sketch of what must always form an interesting chapter in the history of nitrous oxide. In furnishing it I have had no desire to claim for myself any more credit than properly belongs to me, for the interest which I have taken in what I believed to be a valuable application, and for the personal efforts I have made to direct to it the attention of a somewhat prejudiced public. To all who have aided me I shall always be ready to give the praise they have severally merited. To Dr. Colton in particular I have been indebted, who when in Europe, cordially co-operated with me, and whose extended experience with the gas in the United States, rightfully entitled him to be considered as an authority in every thing which concerned its anæsthetic administration.

Yours very truly,

THOMAS W. EVANS, M.D.

Surgeon Dentist to the Emperor.

